# Effect of vitamin D supplementation on type 2 diabetes biomarkers: an umbrella of interventional meta-analyses

**DOI:** 10.1186/s13098-023-01010-3

**Published:** 2023-04-19

**Authors:** Vali Musazadeh, Zeynab Kavyani, Naghmeh Mirhosseini, Parvin Dehghan, Mahdi Vajdi

**Affiliations:** 1 Student Research Committee, Tabriz University of Medical Sciences, Tabriz, Iran; 2 School of Nutrition and Food Sciences, Tabriz University of Medical Sciences, Tabriz, Iran; 3 School of Public Health, University of Saskatchewan, Saskatoon, SK Canada; 4 Associate of Nutrition, School of Nutrition and Food Sciences, Tabriz University of Medical Sciences, Tabriz, Iran; 5 Immunology Research Center, Tabriz University of Medical Sciences, Tabriz, Iran; 6 Student Research Committee, Department of Community Nutrition, School of Nutrition and Food Science, Isfahan University of Medical Sciences, Isfahan, Iran

**Keywords:** Vitamin D, Glycemic indices, Diabetes, Umbrella meta-analysis

## Abstract

**Background:**

Vitamin D supplementation exerts several supporting effects on improving glycemic status, however, results are inconclusive. Thus, in the present study, we aimed to conduct an umbrella of meta-analysis regarding the impact of vitamin D on type 2 diabetes (T2DM) biomarkers.

**Methods:**

The Scopus, PubMed, Web of Science, Embase, and Google Scholar online databases were searched up to March 2022. All meta-analyses evaluating the impact of vitamin D supplementation on T2DM biomarkers were considered eligible. Overall, 37 meta-analyses were included in this umbrella meta-analysis.

**Results:**

Our findings indicated that vitamin D supplementation significantly decreased fasting blood sugar (FBS) (WMD =  − 3.08; 95% CI: − 3.97, − 2.19, p < 0.001, and SMD =  − 0.26; 95% CI: − 0.38, − 0.14, p < 0.001), hemoglobin A1c (HbA1c) (WMD =  − 0.05; 95% CI: − 0.10, − 0.01, p = 0.016, and SMD =  − 0.16; 95% CI: − 0.27, − 0.05, p = 0.004), insulin concentrations (WMD =  − 2.62; 95% CI: − 4.11, − 1.13; p < 0.001, and SMD =  − 0.33; 95% CI: − 0.56, − 0.11, p = 0.004), and homeostatic model assessment for insulin resistance (HOMA-IR) (WMD =  − 0.67; 95% CI: − 1.01, − 0.32, p < 0.001, and SMD =  − 0.31; 95% CI: − 0.46, − 0.16, p < 0.001).

**Conclusion:**

This umbrella meta-analysis proposed that vitamin D supplementation may improve T2DM biomarkers.

**Supplementary Information:**

The online version contains supplementary material available at 10.1186/s13098-023-01010-3.

## Background

Impaired glucose metabolism is associated with an increased risk of several chronic diseases, including obesity, Type 2 diabetes (T2DM), metabolic syndrome, and cardiovascular disease [1]. Both genetic predispositions and unhealthy lifestyles might incorporate into hyperglycemic complications. The actual genetic origin of hyperglycemia has not yet been identified, however there is robust evidence that obesity, unhealthy eating patterns, and sedentary lifestyles are the main modifiable non-genetic risk factors [2, 3]. Although one of the most important first-line treatments for hyperglycemia is dietary modification, however, their effectiveness is modest [4, 5]. Recently, nutritional adjuvant therapies, such as chromium [6], magnesium [7], omega-3 fatty acids [8], and vitamin C [9] have been given more attention due to the adverse effects of pharmacological treatments. Among others, vitamin D has been well studied in clinical practice for its therapeutic effects [10, 11].

Vitamin D, a lipid-soluble vitamin, is well-known for regulating bone metabolism and calcium-phosphorus homeostasis [11]. However, it exerts a variety of non-skeletal benefits, mainly managing different chronic diseases as well [12, 13]. Vitamin D deficiency is involved in abnormal glucose metabolism, altered insulin secretion and T2DM [14]. Vitamin D deficiency is very prevalent among patients with T2DM [15]. Mitri et al. [16] found that even a slight increase in vitamin D intake [from < 5 µgr/day (200 IU/days) to 12.5 µgr/day (> 500 IU/days)] reduced the risk of T2DM by 13%. Vitamin D deficiency in T2DM patients might impair insulin secretion leading to abnormal glucose metabolism and insulin resistance [17, 18]. Moreover, several studies have reported the hypoglycemic properties of vitamin D [19–21]. Vitamin D protects against diabetes-related complications through its antioxidant, anti-inflammatory, and immune-modulating effects which plays an important role in insulin resistance [11]. The positive benefits of vitamin D on glycemic control have been revealed in several human studies of diabetes [22–24]. Also, there are evidence supporting that vitamin D could decrease lipid concentrations, improve immune regulation, and reduce oxidative stress [25, 26].

The impact of vitamin D on T2DM biomarkers has been broadly examined through many meta-analyses of randomized controlled trials (RCTs), yet the fact that vitamin D supplementation is an effective strategy for controlling T2DM still remains controversial, which has led to inconsistent conclusions about the role of vitamin D on T2DM biomarkers [10, 27–29]. Therefore, the current study was designed as an umbrella meta-analysis to investigate the summarized effects of supplementation with vitamin D on T2DM biomarkers found by previous meta-analyses with the aim of addressing the inconsistency among current evidence.

## Methods

The current umbrella review of meta-analysis, was performed in adherence to the Preferred Reporting Items for Systematic Reviews and Meta-Analyses (PRISMA) statement [30], and the protocol was registered in PROSPERO (Registration ID: CRD42021292700).

### .Search strategy

A comprehensive online search for relevant published records was conducted from inception until March 2022, using Scopus, Web of Science databases, Embase, PubMed, and Google Scholar. Based on MeSH and text keywords, the following pattern of search was applied: "vitamin d" OR "ergocalciferols" OR "supplementation "OR "vitamin d3″ OR "vitamin d2″ OR "intake" AND “blood glucose” OR “Glucose” OR “FBS” OR “HOMA-IR” OR “insulin sensitivity” OR Insulin” OR “HbA1c” OR “insulin resistance” AND "systematic review" OR "meta-analysis". To enhance the sensitivity of the search approach, the wild-card phrase "*" was used. Database searches were done by two authors (VM and MV). Hand searches were also conducted on the reference lists of related articles to ensure that no studies were missed. We included English-language publications.

### Study selection

Meta-analyses investigating the effect of vitamin D supplementation on T2DM biomarkers (FBS, HbA1c, insulin, and HOMA-IR) providing the effect sizes (ESs) and confidence intervals (CIs) were considered eligible for including in this umbrella meta-analysis of randomized controlled trials (RCTs). Studies with the following criteria were excluded: observational studies, quasi-experimental studies, case reports, conference papers, letters, in *vitro*, in *vivo*, and ex vivo studies, controlled clinical trials, studies with insufficient data, and studies without full texts. The paper selection process was completed by two independent reviewers (ZK and VM), and any disagreements came into a consensus through discussing with a senior author (PD).

### Data extraction

Two independent reviewers (ZK, and MV) extracted the following information from included studies: the first author, publication year, location of the project, study population and sample size, dosage and duration range of Vitamin D, ESs and CIs [(standardized mean difference (SMD), and weighted mean difference (WMD)] regarding study outcomes. The disagreements were consulted by a third reviewer (VM).

### Quality assessment

The methodological quality of eligible articles was assessed by two independent reviewers using the assessment of multiple systematic reviews (AMSTAR2) tool (VM, and MV). The AMSTAR2 questionnaire consists of 16 questions, which reviewers are required to answer "Yes," "Partial Yes", "No", or "No Meta-analysis". “High quality”, “Moderate quality”, “Low quality”, and “Critically low quality” were the categories on the AMSTAR2 checklist [31].

### Statistical analysis

Random-effect models, based on the restricted maximum likelihood method (REML), were used to estimate the overall ESs and 95% CI [32]. Heterogeneity across studies was estimated by Cochran Q and I^2^ statistics, in which I^2^ values greater than 50% or p < 0.1 were considered as significant heterogeneity. A separate analysis was carried out for each type of SMD and WMD in view of their natural differences. In order to explore sources of heterogeneity, we performed subgroup analysis applying the duration of study (≤ 15, and > 15 weeks), gender (Women, both), mean age (≤ 50, and > 50 years), sample size (≤ 500, 500–1000, and > 1000), dose (≤ 4000, and > 4000 IU/day), and health conditions (GDM, PCOS, NAFLD, obesity, diabetic nephropathy, prediabetes, and dialysis patients). The sensitivity analysis was conducted to establish how dependent the overall ES was on a specific study (Leave-one-out Method). Egger's and Begg's tests were used to examine the small-study effect. The presence of publication bias was detected using a visual inspection of the funnel plot. If publication bias was identified, the trim and fill method carried out. STATA version 16 software was used for the statistical analyses (Stata Corp, College Station, Texas, USA).

## Results

### Selected studies and systematic review

**Fig. 1:**
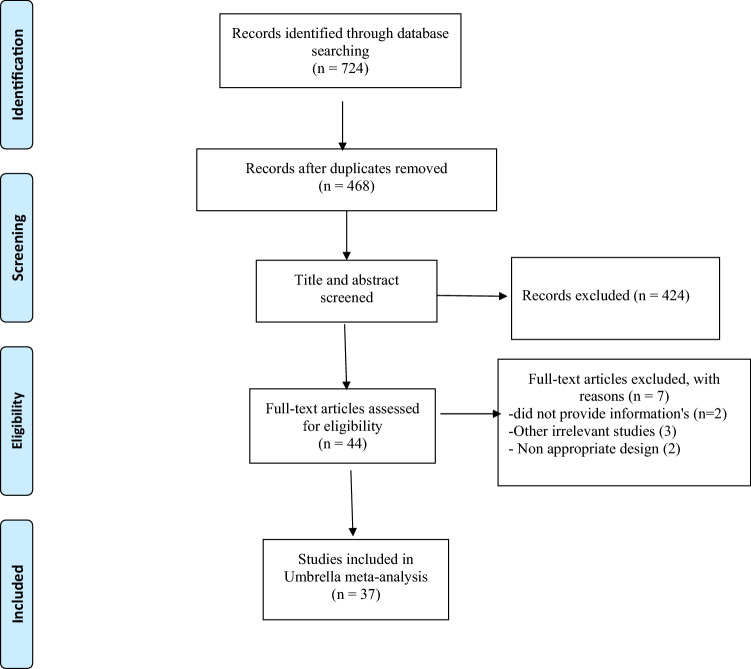
Flow chart of study selection

The PRISMA flow chart of the literature search process is depicted in Fig. 1. Through electronic database searches, 724 articles were initially identified, of which 246 were duplicates. After reviewing the titles and abstracts of 468 studies, 424 articles did not meet the inclusion criteria, so they were excluded from any further analysis. Eventually, 37 meta-analyses published between 2011 and 2021 were qualified to be included in the umbrella review. The characteristics of the included meta-analyses are listed in Table 1. The age range of 38,000 participants included in the current study was between 26 and 60 years with the mean of 44.7 years. Intervention duration ranged between 7 and 47 weeks.

**Table 1:** Study characteristics of included studie

Citation (First author et al.)	Year	Location	No. of participants in meta-analysis	Gender/age (years)	Health condition	Dose (IU/d)	Duration (week)
Akbari et al.	2017	Iran	371	Women /NR	GDM	NR	7.5
Ojo et al.	2019	UK	359	Women /30	GDM	2774	8.5
Guo et al.	2020	China	730	Women /31	PCOS	545.5	12
Rezaei et al.	2021	Iran	686	Both /NR	NAFLD	21000	15.5
Sarathy et al.	2014	Iran	131	Both /45	dialysis patients	NR	8
Tabrizi et al.	2017	Iran	332	Both /46	NAFLD	7000	12
Zou (a) et al. Zou (b) et al.	2021	China	639 538	Both/55 Both/60	Diabetes Prediabetes	2700 1500	12.5 25
Wei et al.	2020	China	468	Both /46	NAFLD	10000	20
Wang et al.	2021	China	389	Women /26	PCOS	5000	13
Wang et al.	2019	China	230	Both /51	DN	12	12
Zhang et al.	2021	China	1486	Both /50	Prediabetes	5483	47
Wu et al.	2017	China	1496	Both /56	TD2M	1970	19
Elamin et al.	2011	USA	2081	Both /NR	Elderly people with different diseases	NR	NR
Sahebi (a) et al. Sahebi (b) et al.	2018	Iran	NR	Women /NR	GDM T2DM	NR	NR
Tang et al.	2018	USA	5509	Both /NR	Diabetic Adults	NR	NR
Mirhosseini et al.	2018	Canada	3062	Both /49	Prediabetics	4030	NR
Milajerdi et al.	2019	Iran	214	Both /50	CKD	2683	8
Li et al.	2018	China	2104	Both /56	T2DM	4991	18
Lee et al.	2017	USA	2295	Both/54	T2DM	3410	40
Łagowska et al.	2018	Poland	458	Women/26	PCOS	4614	16
Krul-Poel et al.	2017	Netherlands	1180	Both /NR	T2DM	4047	24
Wang et al.	2020	China	717	Women /26	GDM	1884	7
Jamka et al.	2015	Poland	590	Both /NR	Overweight/obese	3047	20
Jahanjoo et al.	2018	Iran	223	Both/30	GDM	2976	10
He et al.	2018	China	NR	Both/30	Diabetics	NR	NR
Emadzadeh et al.	2020	Iran	722	Both/46	Different diseases	4637	17
Gasparri et al.	2019	Italy	339	Both /53	Different diseases (GDM & T2DM & MetS)	875	12
Mirhosseini (a) et al. Mirhosseini (b) et al.	2017	Canada	330 1331	Both /51 Both /48	Obese diabetic Non-obese diabetic	4243 3385	32 13
Ostadmohammadi et al.	2019	Iran	630	Both /NR	CVD	NR	NR
Guo et al.	2020	China	413	Both /45	NAFLD	2878	23
Pramono et al.	2020	Netherlands	1220	Both /NR	Diabetes	4600	15
Seida (a) et al. Seida (b) et al. Seida (c) et al.	2014	Boston	742 309 471	Both /51 Both /55 Both /55	Normal glucose tolerance Prediabetes Established T2DM	4920 6238 3437	42 26 17
Hu (a) et al. Hu (b) et al.	2019	China	375 1059	Both /NR	T2DM	3782 5030	29 (long term) 14 (short term)
Poolsup et al.	2015	Thailand	537	Both /53	prediabetes	6600	40
MIAO et al.	2020	China	278	Both /NR	PCOS	NR	NR
Manousopoulou et al.	2015	UK	787	Both /40	Obesity	3095	26
Gallo et al.	2019	NR	366	Women /29	Pregnancy	3413	9

NR, Not reported; T2DM, type 2 diabetes mellitus; GDM, gestational diabetes mellitus; DN, diabetic nephropathy; CVD, Cardiovascular disease; PCOS, polycystic ovary syndrome; NAFLD, Non-Alcoholic Fatty Liver; MetS, metabolic syndrome; HD, hemodialysis; CKD, Chronic kidney disease

Regarding study location, fourteen meta-analyses were performed in China [10, 27, 33–44], nine in Iran [19, 28, 45–51], four in the USA [52–55], two in UK [21, 56], two in Canada [11, 57], two in Poland [29, 58], two in Netherlands [59, 60], one in Italy [20], and one in Thailand [61]. Cochrane risk of bias tool was used for quality assessment. Overall, almost all randomized controlled trials (RCTs) qualified in the meta-analyses were of high quality. Detailed information is presented in Table 1 about the quality of the RCTs in the meta-analyses.

### Methodological quality assessment

Table 2 presents the findings of the quality assessment of meta-analyses according to the AMSTAR2 questionnaire.

**Table 2:** The results of the methodological quality assessment of the meta-analysis

Citation (First author et al.)	Year	Q1^1^	Q2	Q3	Q4	Q5	Q6	Q7	Q8	Q9	Q10	Q11	Q12	Q13	Q14	Q15	Q16	Quality assessment
Akbari et al	2017	Yes	Yes	Yes	Partial Yes	Yes	Yes	Yes	Yes	Yes	No	Yes	No	Yes	Yes	Yes	Yes	High
Ojo et al	2019	Yes	Yes	Yes	Yes	No	Yes	Yes	Yes	Yes	Yes	Yes	Yes	Yes	Yes	Yes	Yes	High
Guo et al	2020	No	Yes	Yes	Partial Yes	Yes	Yes	Yes	Yes	Yes	Yes	Yes	No	Yes	No	Yes	Yes	Moderate
Rezaei et al	2021	Yes	Yes	Yes	Yes	Yes	Yes	Yes	Yes	Yes	Yes	Yes	Yes	Yes	Yes	Yes	Yes	High
Sarathy et al	2014	No	Yes	No	No	Yes	Yes	Yes	No	Yes	Yes	Yes	Yes	No	No	No	Yes	Low
Tabrizi et al	2017	No	Yes	Yes	Yes	Yes	Yes	Yes	No	Yes	No	Yes	Yes	No	Yes	Yes	Yes	Moderate
Zou et al	2021	No	Yes	Yes	Partial Yes	Yes	Yes	No	Yes	Yes	No	Yes	Yes	No	No	Yes	Yes	Moderate
Wei et al	2020	No	Yes	Yes	No	No	Yes	Yes	No	Yes	Yes	Yes	Yes	No	Yes	Yes	Yes	Moderate
Wang et al	2021	No	Yes	Yes	Yes	Yes	Yes	Yes	Yes	Yes	Yes	Yes	Yes	Yes	Yes	Yes	Yes	High
Wang et al	2019	No	Partial Yes	Yes	Partial Yes	Yes	Yes	Yes	Yes	Yes	Yes	Yes	Yes	No	Yes	Yes	Yes	High
Zhang et al	2021	No	Partial Yes	Yes	Yes	Yes	Yes	Yes	No	Yes	Yes	Yes	Yes	No	Yes	Yes	Yes	High
Wu et al	2017	No	Yes	Yes	No	No	Yes	Yes	No	No	Yes	Yes	No	No	Yes	Yes	Yes	Low
Elamin et al	2011	No	Partial Yes	Yes	Partial Yes	Yes	Yes	Yes	Yes	No	No	Yes	No	No	Yes	No	No	Low
Sahebi et al	2018	No	Partial Yes	Yes	Partial Yes	Yes	Yes	Yes	Yes	Yes	No	Yes	Yes	No	Yes	No	Yes	Moderate
Tang et al	2018	No	Yes	Yes	Yes	No	Yes	Yes	Yes	Yes	No	Yes	No	No	Yes	Yes	Yes	Moderate
Mirhosseini et al	2018	No	Yes	Yes	Partial Yes	Yes	Yes	Yes	Yes	Yes	Yes	Yes	Yes	Yes	Yes	Yes	Yes	High
Milajerdi et al	2019	No	Yes	Yes	Yes	Yes	Yes	Yes	Yes	Yes	Yes	Yes	Yes	No	Yes	Yes	Yes	High
Li et al	2018	No	Yes	Yes	Yes	Yes	Yes	Yes	Yes	Yes	Yes	Yes	Yes	No	Yes	No	Yes	High
Lee et al	2017	No	Partial Yes	Yes	Yes	Yes	Yes	Yes	No	Yes	Yes	Yes	Yes	No	Yes	Yes	Yes	Moderate
Łagowska et al	2018	No	Partial Yes	Yes	Yes	Yes	Yes	Yes	Yes	Yes	No	Yes	No	No	Yes	No	Yes	Moderate
Krul-Poel et al	2017	No	Partial Yes	Yes	Partial Yes	Yes	Yes	Yes	Yes	Yes	No	Yes	Yes	No	Yes	Yes	Yes	Moderate
Wang et al	2020	No	Partial Yes	Yes	Yes	Yes	Yes	Yes	Yes	Yes	Yes	Yes	Yes	Yes	Yes	No	Yes	High
Jamka et al	2015	No	Yes	Yes	Yes	No	Yes	Yes	Yes	Yes	No	Yes	No	No	Yes	No	Yes	Moderate
Jahanjoo et al	2018	Yes	Yes	Yes	Partial Yes	Yes	Yes	Yes	Yes	Yes	No	Yes	Yes	No	Yes	Yes	No	Moderate
He et al	2018	No	Partial Yes	Yes	Partial Yes	Yes	Yes	Yes	Yes	Yes	Yes	Yes	Yes	No	Yes	Yes	Yes	High
Emadzadeh et al	2020	No	Yes	Yes	Partial Yes	Yes	Yes	Yes	Yes	Yes	No	Yes	Yes	Yes	Yes	No	Yes	Moderate
Gasparri et al	2019	No	Partial Yes	Yes	Yes	Yes	Yes	Yes	No	Yes	No	Yes	No	No	Yes	Yes	No	Moderate
Mirhosseini et al	2017	No	Partial Yes	Yes	Yes	Yes	Yes	Yes	Yes	Yes	No	Yes	Yes	Yes	Yes	Yes	Yes	High
Ostadmohammadi et al	2019	No	No	No	Yes	Yes	Yes	No	No	Yes	No	No	Yes	No	Yes	No	No	Critically low
Guo et al	2020	Yes	Partial Yes	Yes	Yes	Yes	Yes	Yes	Yes	Yes	Yes	Yes	Yes	Yes	Yes	Yes	Yes	High
Pramono et al	2020	No	Yes	Yes	Partial Yes	Yes	Yes	Yes	Yes	Yes	No	Yes	Yes	Yes	Yes	Yes	Yes	High
Seida et al	2014	No	Partial Yes	Yes	Partial Yes	Yes	Yes	Yes	Yes	Yes	No	Yes	Yes	Yes	Yes	Yes	No	Moderate
Hu et al	2019	No	Yes	Yes	Yes	Yes	Yes	No	Yes	No	No	Yes	Yes	No	Yes	No	No	Low
Poolsup et al	2015	No	Partial Yes	Yes	Yes	Yes	Yes	Yes	Yes	Yes	No	Yes	No	No	Yes	Yes	Yes	Moderate
MIAO et al	2020	No	Partial Yes	Yes	Partial Yes	Yes	Yes	Yes	No	Yes	No	Yes	Yes	Yes	Yes	Yes	Yes	Moderate
Manousopoulou et al	2015	No	Yes	Yes	Partial Yes	Yes	Yes	Yes	Yes	Yes	No	Yes	Yes	No	Yes	No	Yes	Moderate
Gallo et al	2019	No	Partial Yes	Yes	Partial Yes	Yes	Yes	Yes	Yes	No	Yes	Yes	Yes	No	Yes	Yes	Yes	Moderate

^* 1. Did the research questions and inclusion criteria for the review include the components of PICO? 2. Did the report of the review contain an explicit statement that the review methods were established prior to the conduct of the review and did the report justify any significant deviations from the protocol? 3. Did the review authors explain their selection of the study designs for inclusion in the review? 4. Did the review authors use a comprehensive literature search strategy? 5. Did the review authors perform study selection in duplicate? 6. Did the review authors perform data extraction in duplicate? 7. Did the review authors provide a list of excluded studies and justify the exclusions? 8. Did the review authors describe the included studies in adequate detail? 9. Did the review authors use a satisfactory technique for assessing the risk of bias (RoB) in individual studies that were included in the review? 10. Did the review authors report on the sources of funding for the studies included in the review? 11. If meta^^−^^analysis was performed, did the review authors use appropriate methods for statistical combination of results? 12. If meta^^−^^analysis was performed, did the review authors assess the potential impact of RoB in individual studies on the results of the meta^^−^^analysis or other evidence synthesis? 13. Did the review authors account for RoB in individual studies when interpreting/ discussing the results of the review? 14. Did the review authors provide a satisfactory explanation for, and discussion of, any heterogeneity observed in the results of the review? 15. If they performed quantitative synthesis, did the review authors carry out an adequate investigation of publication bias (small study bias) and discuss its likely impact on the results of the review? 16. Did the review authors report any potential sources of conflict of interest, including any funding they received for conducting the review?^

^Each question was answered with “Yes”, “Partial Yes” or “No”. When no meta^^−^^analysis was done, question 11, 12 and 15 were answered with “No meta^^−^^analysis conducted^

### Effects of vitamin D on FBS

#### According to WMD analysis

**Fig. 2:**
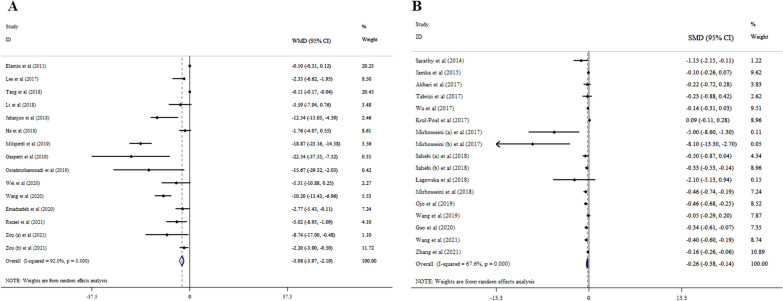
Forest plot with mean difference and 95% confidence intervals (CIs), the effects of vitamin D supplementation on FBS levels according to WMD (**A**), and SMD (**B**) analysis

The results of 14 eligible studies with 15 ESs, including 17,136 participants revealed that supplementation with vitamin D significantly decreased FBS (WMD = − 3.08; 95% CI: − 3.97, − 2.19, p < 0.001) (Fig. 2A). A significant heterogeneity was detected among meta-analyses (*I*^*2*^ = 92.0%, p < 0.001). Subgroup analyses indicated that the reductions in FBS levels were more pronounced in patients with a mean age of > 50 years, patients with gestational diabetes mellitus (GDM), a sample size of ≤ 1000, and studies with a duration of intervention ≤ 15 weeks, and dosage of ≤ 4000 IU/day when compared to their counterparts (Table 3).

**Table 3:** Pooled estimates of vitamin D on T2DM biomarkers according to WMD analysis

Group	No. of comparisons	WMD (95% CI)	P-value	*I*^*2*^ (%)	P-heterogeneity
*Vit D supplementation on FBS levels*					
Total	15	− 3.08 (− 3.97, − 2.19)	< 0.001	92..0	< 0.001
*Sample size*					
≤ 500	4	− 13.77 (− 20.92, − 6.63)	< 0.001	81.0	< 0.001
500–1000	6	− 5.79 (− 9.08, − 2.49)	< 0.001	78.8	< 0.001
> 1000	4	− 0.13 (− 0.35, 0.08)	0.226	49.5	0.115
NR	1	− 1.76 (− 4.07, 0.55)	0.135	–	–
*Age (year)*					
≤ 50	4	− 7.54 (− 12.31, − 2.77)	0.002	88.0	< 0.001
> 50	6	− 8.09 (− 13.38, − 2.80)	0.003	91.6	< 0.001
NR	5	− 0.19 (− 0.53, 0.16)	0.285	69.1	0.011
*Health condition*					
GDM	2	− 10.83 (− 13.60, − 8.07)	< 0.001	0.0	0.461
CVD	1	− 15.67 (− 29.32, − 2.03)	0.024	–	–
NAFLD	2	− 5.12 (− 8.33, − 1.91)	0.002	0.0	0.934
Diabetes	6	− 1.86 (− 3.42, − 0.29)	0.020	72.0	0.003
CKD	1	− 18.87 (− 23.16, − 14.58)	< 0.001	–	–
Different diseases	2	− 11.22 (− 30.39, 7.95)	0.251	84.5	0.011
Elderly people with different diseases	1	− 0.10 (− 0.31, 0.11)	0.362	–	–
*Duration (week)*					
≤ 15	5	− 13.62 (− 18.18, − 9.05)	< 0.001	68.4	0.013
> 15	6	− 2.76 (− 3.87, − 1.66)	< 0.001	0.0	0.736
NR	4	− 0.13 (− 0.37, 0.12)	0.301	56.9	0.073
*Dose (IU/day)*					
≤ 4000	7	− 9.86 (− 14.90, − 4.81)	< 0.001	92.4	< 0.001
> 4000	4	− 3.70 (− 5.55, − 1.85)	< 0.001	0.0	0.747
NR	4	− 0.13 (− 0.37, 0.12)	0.301	56.9	0.073
*Vit D supplementation on HbA1c levels*					
Total	11	− 0.05 (− 0.10, − 0.01)	0.016	50.4	0.028
*Sample size*					
≤ 500	3	− 0.09 (− 0.19, − 0.00)	0.040	0.0	0.401
500–1000	5	− 0.02 (− 0.07, 0.03)	0.390	40.0	0.155
> 1000	3	− 0.14 (− 0.31, 0.03)	0.105	71.4	0.030
*Age (year)*					
≤ 50	2	− 0.09 (− 0.36, 0.19)	0.537	60.7	0.111
> 50	8	− 0.08 (− 0.15, − 0.01)	0.019	58.6	0.018
NR	1	− 0.04 (− 0.07, − 0.00)	0.025	–	–
*Health condition*					
diabetes	6	− 0.06 (− 0.13, 0.00)	0.063	63.4	0.018
prediabetes	2	− 0.07 (− 0.12, − 0.01)	0.015	0.0	0.733
CKD	1	− 0.69 (− 1.71, 0.33)	0.185	–	–
Diabetic Nephropathy	1	0.01 (− 0.07, 0.09)	0.806	–	–
different diseases	1	− 0.29 (− 0.65, 0.07)	0.114	–	–
*Duration (week)*					
≤ 15	2	− 0.18 (− 0.54, 0.19)	0.339	16.1	0.275
> 15	8	− 0.06 (− 0.13, − 0.00)	0.040	60.2	0.014
NR	1	− 0.04 (− 0.07, − 0.00)	0.025	–	–
*Dose (IU/day)*					
≤ 4000	5	− 0.13 (− 0.23, − 0.03)	0.008	32.3	0.206
> 4000	4	− 0.06 (− 0.16, 0.05)	0.309	57.5	0.070
NR	2	− 0.03 (− 0.07, 0.01)	0.176	20.6	0.262
*Vit D supplementation on HOMA-IR levels*					
Total	17	− 0.67 (− 1.01, − 0.32)	< 0.001	96.2	< 0.001
*Sample size*					
≤ 500	10	− 0.91 (− 1.61, − 0.21)	0.010	95.8	< 0.001
500–1000	4	− 0.72 (− 1.25, − 0.18)	0.009	84.1	< 0.001
> 1000	3	− 0.15 (− 0.43, 0.12)	0.264	78.3	0.010
*Age (year)*					
≤ 50	8	− 1.08 (− 1.78, − 0.37)	0.003	94.5	< 0.001
> 50	5	− 0.05 (− 0.49, 0.40)	0.839	82.5	< 0.001
NR	4	− 0.62 (− 0.99, − 0.25)	< 0.001	76.8	0.005
*Gender*					
Women	2	− 1.08 (− 1.35, − 0.81)	< 0.001	0.0	0.858
Both	15	− 0.59 (− 0.93, − 0.24)	< 0.001	95.6	< 0.001
*Health condition*					
GDM	2	− 1.07 (− 1.40, − 0.73)	< 0.001	0.0	0.876
PCOS	4	− 0.01 (− 0.44, 0.43)	0.975	85.6	< 0.001
NAFLD	2	− 0.21 (− 1.35, 0.94)	0.726	88.9	0.003
Diabetes	4	− 0.23 (− 0.51, 0.05)	0.104	74.7	0.008
CKD	1	− 2.30 (− 2.88, − 1.72)	< 0.001	–	–
Pregnancy	1	− 1.11 (− 1.54, − 0.68)	< 0.001	–	–
CVD	1	− 1.07 (− 1.49, − 0.66)	< 0.001	–	–
different diseases	2	− 2.07 (− 2.74, − 1.40)	< 0.001	0.0	0.504
*Duration (week)*					
≤ 15	6	− 1.57 (− 2.10, − 1.05)	< 0.001	74.0	0.002
> 15	8	− 0.06 (− 0.35, 0.23)	0.694	88.0	< 0.001
NR	3	− 0.58 (− 1.02, − 0.14)	0.009	82.0	0.004
*Dose (IU/day)*					
≤ 4000	10	− 0.98 (− 1.51, − 0.44)	< 0.001	96.8	< 0.001
> 4000	4	− 0.12 (− 0.58, 0.35)	0.615	70.0	0.019
NR	3	− 0.58 (− 1.02, − 0.14)	0.009	82.0	0.004
*Vit D supplementation on Insulin levels*					
Total	9	− 2.62 (− 4.11, − 1.13)	< 0.001	82.2	< 0.001
*Sample size*					
≤ 500	5	− 2.50 (− 6.31, 1.31)	0.199	58.0	0.049
> 500	4	− 3.12 (− 4.72, − 1.52)	< 0.001	87.1	< 0.001
*Age (year)*					
≤ 50	4	− 2.59 (− 5.69, 0.51)	0.102	89.8	< 0.001
> 50	3	− 5.97 (− 13.49, 1.55)	0.120	33.2	0.224
NR	2	− 2.45 (− 4.46, − 0.43)	0.017	91.4	< 0.001
*Health condition*					
GDM	2	− 4.88 (− 6.59, − 3.17)	< 0.001	0.0	0.656
prediabetes	1	− 13.45 (− 25.85, − 1.05)	0.034	–	–
Diabetes	2	− 1.48 (− 2.00, − 0.95)	< 0.001	0.0	0.445
NAFLD	1	0.76 (− 0.53, 2.05)	0.248	–	–
CKD	1	− 2.25 (− 7.18, 2.67)	0.371	–	–
CVD	1	− 3.53 (− 4.59, − 2.46)	< 0.001	–	–
different diseases	1	− 2.94 (− 4.70, − 1.19)	< 0.001	–	–
*Duration (week)*					
≤ 15	3	− 4.60 (− 6.21, − 2.98)	< 0.001	0.0	0.555
> 15	4	− 2.28 (− 6.06, 1.50)	0.237	81.2	< 0.001
NR	2	− 2.45 (− 4.46, − 0.43)	0.017	91.4	< 0.001
*Dose (IU/day)*					
≤ 4000	4	− 4.71 (− 6.44, − 2.98)	< 0.001	3.3	0.376
> 4000	3	− 1.27 (− 4.79, 2.25)	0.479	83.1	0.003
NR	2	− 2.45 (− 4.46, − 0.43)	0.017	91.4	< 0.001

N, Number; NR, not reported

#### According to SMD analysis

The results from 15 meta-analyses with 17 ESs and 12,422 participants reported that vitamin D administration significantly reduced FBS (SMD = − 0.26; 95% CI: − 0.38, − 0.14, p < 0.001), with significant inter-study heterogeneity (*I*^*2*^ = 67.6%, p < 0.001) (Fig. 2B). Conducting subgroup analysis indicated that the effects of vitamin D on FBS were more prominent among women and the sample size ≤ 500, intervention duration of ≤ 15 weeks, patients with GDM and polycystic ovary syndrome (PCOS), and subjects with the mean age of ≤ 50 years than the entire sample (Table 4).

**Table 4:** Pooled estimates of Vitamin D on T2DM biomarkers according to SMD analysis

Group	No. of comparisons	SMD (95% CI)	P-value	*I*^*2*^ (%)	P-heterogeneity
*Vit D supplementation on FBS levels*					
Total	17	− 0.26 (− 0.38, − 0.14)	< 0.001	67.6	< 0.001
*Sample size*					
≤ 500	8	− 0.36 (− 0.60, − 0.12)	0.004	59.0	0.017
500–1000	2	− 0.20 (− 0.43, 0.03)	0.096	54.7	0.137
> 1000	5	− 0.17 (− 0.38, 0.05)	0.123	79.3	0.001
NR	2	− 0.36 (− 0.54, − 0.18)	< 0.001	0.0	0.501
*Age (year)*					
≤ 50	8	− 0.44 (− 0.62, − 0.26)	< 0.001	41.7	0.100
> 50	4	− 0.14 (− 0.31, 0.03)	0.116	59.8	0.059
NR	5	− 0.17 (− 0.36, 0.02)	0.086	65.8	0.020
*Gender*					
Women	7	− 0.38 (− 0.49, − 0.28)	< 0.001	0.0	0.835
Both	10	− 0.17 (− 0.33, − 0.01)	0.035	70.6	< 0.001
*Health condition*					
GDM	3	− 0.43 (− 0.62, − 0.25)	< 0.001	0.0	0.657
PCOS	3	− 0.38 (− 0.55, − 0.22)	< 0.001	0.0	0.508
NAFLD	1	− 0.23 (− 0.88, 0.42)	0.488	–	–
T2DM and Non-obese	4	− 0.15 (− 0.46, 0.16)	0.330	83.0	< 0.001
Overweight and Obese	2	− 2.20 (− 6.95, 2.55)	0.365	85.5	0.009
Dialysis patients	1	− 1.13 (− 2.15, − 0.11)	0.030	–	–
Prediabetics	2	− 0.28 (− 0.57, 0.01)	0.056	75.2	0.044
Diabetic Nephropathy	1	− 0.05 (− 0.29, 0.19)	0.689	–	–
*Duration (week)*					
≤ 15	8	− 0.34 (− 0.55, − 0.13)	< 0.001	60.6	0.013
> 15	6	− 0.10 (− 0.25, 0.05)	0.196	63.4	0.018
NR	3	− 0.39 (− 0.54, − 0.24)	< 0.001	0.0	0.658
*Dose (IU/day)*					
≤ 4000	6	− 0.22 (− 0.42, − 0.03)	0.024	73.0	0.002
> 4000	7	− 0.25 (− 0.48, − 0.02)	0.030	75.2	< 0.001
NR	4	− 0.36 (− 0.53, − 0.20)	< 0.001	0.0	0.400
*Vit D supplementation on HbA1c levels*					
Total	13	− 0.16 (− 0.27, − 0.05)	0.004	74.0	< 0.001
*Sample size*					
≤ 500	4	− 0.12 (− 0.34, 0.11)	0.310	54.8	0.084
500–1000	2	− 0.16 (− 0.50, 0.18)	0.364	92.1	< 0.001
> 1000	5	− 0.14 (− 0.28, 0.00)	0.052	77.7	< 0.001
NR	2	− 1.07 (− 1.71, − 0.42)	< 0.001	0.0	0.815
*Age (year)*					
≤ 50	2	− 0.35 (− 0.49, − 0.20)	< 0.001	0.0	0.858
> 50	5	− 0.15 (− 0.28, − 0.02)	0.024	68.9	0.012
NR	6	− 0.09 (− 0.31, 0.13)	0.414	75.0	< 0.001
*Gender*					
Women	4	− 0.47 (− 0.91, − 0.03)	0.035	49.2	0.117
Both	9	− 0.12 (− 0.23, − 0.02)	0.024	76.3	< 0.001
*Health condition*					
GDM	3	− 0.31 (− 0.59, − 0.03)	0.029	5.0	0.349
T2DM and Non-obese	6	− 0.16 (− 0.35, 0.02)	0.088	83.4	< 0.001
Prediabetics	2	− 0.26 (− 0.63, 0.11)	0.165	81.1	0.019
Diabetic Nephropathy	1	0.01 (− 0.09, 0.11)	0.845	–	–
obese	1	− 0.16 (− 0.45, 0.13)	0.280	–	–
*Duration (week)*					
≤ 15	4	− 0.25 (− 0.38, − 0.12)	< 0.001	34.7	0.204
> 15	6	− 0.04 (− 0.14, 0.06)	0.476	63.1	0.019
NR	3	− 0.67 (− 1.09, − 0.25)	0.002	24.9	0.264
*Dose (IU/day)*					
≤ 4000	7	− 0.15 (− 0.32, 0.01)	0.069	83.2	< 0.001
> 4000	3	− 0.12 (− 0.19, − 0.06)	< 0.001	0.0	0.702
NR	3	− 0.62 (− 1.47, 0.24)	0.157	64.8	0.058
*Vit D supplementation on HOMA− IR levels*					
Total	19	− 0.31 (− 0.46, − 0.16)	< 0.001	75.9	< 0.001
*Sample size*					
≤ 500	9	− 0.44 (− 0.71, − 0.17)	< 0.001	65.4	0.003
500–1000	5	− 0.13 (− 0.35, 0.08)	0.213	68.1	0.014
> 1000	2	− 0.29 (− 0.47, − 0.11)	< 0.001	0.0	0.392
NR	3	− 0.43 (− 0.97, 0.11)	0.118	81.9	0.004
*Age (year)*					
≤ 50	9	− 0.25 (− 0.42, − 0.08)	0.004	53.5	0.028
> 50	2	− 0.43 (− 0.68, − 0.18)	< 0.001	0.0	0.555
NR	8	− 0.33 (− 0.62, − 0.04)	0.027	86.9	< 0.001
*Gender*					
Women	6	− 0.35 (− 0.60, − 0.09)	0.009	54.4	0.052
Both	13	− 0.30 (− 0.48, − 0.12)	< 0.001	80.4	< 0.001
*Health condition*					
GDM	2	− 0.57 (− 0.89, − 0.24)	< 0.001	0.0	0.605
PCOS	3	− 0.17 (− 0.36, 0.02)	0.082	4.6	0.350
Prediabetes	2	− 0.29 (− 0.47, − 0.11)	< 0.001	0.0	0.392
T2DM	7	− 0.40 (− 0.73, − 0.07)	0.019	88.8	< 0.001
NAFLD	2	− 1.43 (− 2.31, − 0.55)	< 0.001	0.0	0.614
Overweight and obese	3	− 0.10 (− 0.23, 0.03)	0.116	0.0	0.442
*Duration (week)*					
≤ 15	8	− 0.44 (− 0.72, − 0.17)	0.002	68.5	0.002
> 15	7	− 0.15 (− 0.32, 0.02)	0.087	57.1	0.030
NR	4	− 0.38 (− 0.74, − 0.03)	0.035	80.3	0.002
*Dose (IU/day)*					
≤ 4000	8	− 0.37 (− 0.64, − 0.10)	0.007	84.4	< 0.001
> 4000	7	− 0.21 (− 0.37, − 0.05)	0.009	21.6	0.264
NR	5	− 0.48 (− 0.94, − 0.02)	0.039	81.8	< 0.001
*Vit D supplementation on Insulin levels*					
Total	12	− 0.33 (− 0.56, − 0.11)	0.004	81.8	< 0.001
*Sample size*					
≤ 500	8	− 0.80 (− 1.58, − 0.02)	0.045	82.6	< 0.001
> 500	2	− 0.24 (− 0.59, 0.11)	0.185	82.5	0.017
NR	2	− 0.12 (− 0.34, 0.09)	0.273	87.6	0.005
*Age (year)*					
≤ 50	8	− 0.57 (− 1.01, − 0.13)	0.010	83.8	< 0.001
> 50	1	− 0.84 (− 1.67, − 0.00)	0.049	–	–
NR	3	− 0.03 (− 0.12, 0.06)	0.497	0.0	0.718
*Gender*					
Women	5	− 0.85 (− 1.69, − 0.02)	0.046	86.7	< 0.001
Both	7	− 0.20 (− 0.39, 0.00)	0.053	74.7	< 0.001
*Health condition*					
GDM	2	− 2.13 (− 5.90, 1.64)	0.268	95.5	< 0.001
PCOS	3	− 0.29 (− 0.62, 0.05)	0.091	26.6	0.256
Diabetes	2	− 0.32 (− 1.10, 0.47)	0.428	73.2	0.053
NAFLD	2	− 1.04 (− 2.14, 0.05)	0.061	49.6	0.159
Dialysis Patients	1	1.32 (− 0.15, 2.79)	0.078	–	–
Overweight and Obese	1	− 0.07 (− 0.23, 0.09)	0.406	–	–
Prediabetes	1	− 0.23 (− 0.34, − 0.13)	< 0.001	–	–
*Duration (week)*					
≤ 15	7	− 0.75 (− 1.46, − 0.04)	0.039	84.9	< 0.001
> 15	5	− 0.15 (− 0.30, 0.01)	0.064	68.4	0.013
*Dose (IU/day)*					
≤ 4000	7	− 0.47 (− 0.80, − 0.14)	0.005	86.6	< 0.001
> 4000	4	− 0.13 (− 0.55, 0.29)	0.543	46.0	0.135
NR	1	− 0.25 (− 1.02, 0.52)	0.527	–	–

N; Number, NR; not reported

### Effects of vitamin D on HbA1c

#### According to WMD analysis

**Fig. 3:**
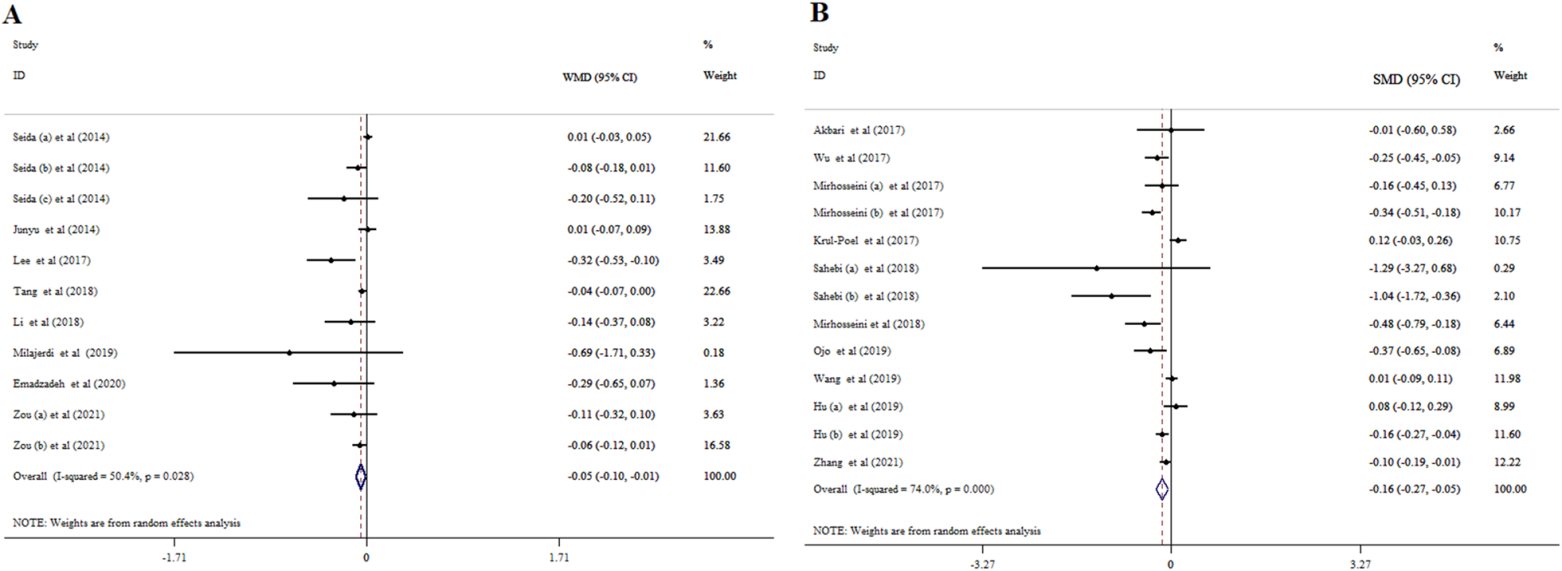
Forest plot with mean difference and 95% confidence intervals (CIs), the effects of vitamin D supplementation on HbA1c levels according to WMD (**A**), and SMD (**B**) analysis

Overall, eight meta-analyses with 11 ESs (11,139 subjects) indicated that vitamin D administration significantly improved HbA1c (WMD = − 0.05; 95% CI: − 0.10, − 0.01, p = 0.016) with a high degree of study heterogeneity (*I*^*2*^ = 50.4%, p = 0.401) (Fig. 3A). Subgroup analysis revealed that vitamin D with a dosage of ≤ 4000 IU/day and the duration of > 15 weeks for the subjects with prediabetes and the mean age of > 50 years contributed to a robust reduction in HbA1c levels (Table 3).

#### According to SMD analysis

Totally, 10 meta-analyses with 13 ESs, including 11,873 participants, found that supplementation with vitamin D lowered HbA1c significantly (SMD = − 0.16; 95% CI: − 0.27, − 0.05, p = 0.004) (Fig. 3B). The between-study heterogeneity was considerable (*I*^2^ = 74.0%, p < 0.001). The intervention duration of ≤ 15 weeks among women with GDM and age ≤ 50 years contributed to a greater decrease in HbA1c (Table 4).

### Effects of vitamin D on insulin

#### According to WMD analysis

**Fig. 4:**
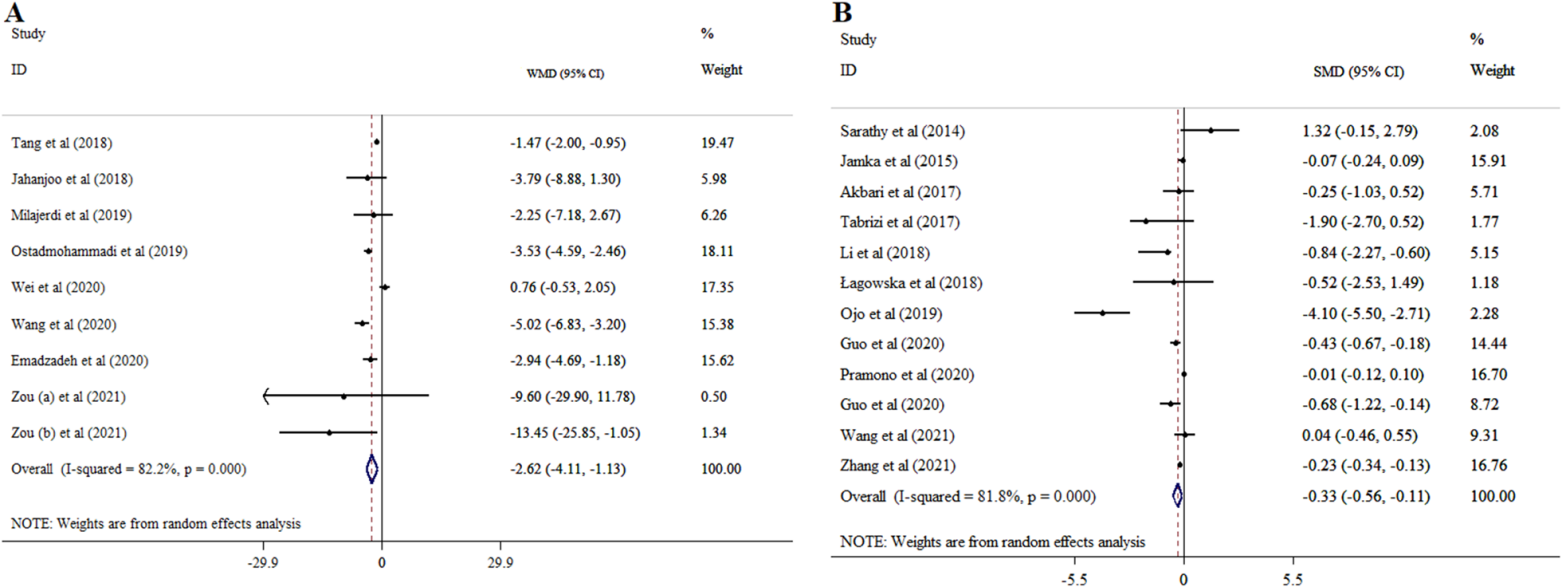
Forest plot with mean difference and 95% confidence intervals (CIs), the effects of vitamin D supplementation on insulin levels according to WMD (**A**), and SMD (**B**) analysis

Finding from eight meta-analyses with nine ESs including 7,723 participants demonstrated that vitamin D substantially decreased insulin level (WMD = − 2.62; 95% CI: − 4.11, − 1.13; p < 0.001) (Fig. 4A) with high heterogeneity between-meta-analyses (*I*^*2*^ = 82.2%, p < 0.001). Vitamin D supplement of ≤ 4000 IU/day in studies with intervention duration of ≤ 15 weeks, subjects younger than 50 years with GDM, sample size of > 500 contributed to a more robust reduction in insulin (Table 3).

#### According to SMD analysis

Results revealed considerable effect of vitamin D supplementation on insulin levels in 12 meta-analyses with 6,118 participants (SMD = − 0.33; 95% CI: − 0.56, − 0.11, p = 0.004; *I*^*2*^ = 81.8%, p < 0.001) (Fig. 4B). From these analyses, we found a significant lowering effect of vitamin D supplementation on insulin in studies with prescribed ≤ 4000 IU/day of vitamin D and treatment duration of ≤ 15 weeks, sample size less than 500 and in women with mean age of ≤ 50 (Table 4).

### Effects of vitamin D on HOMA-IR

#### According to WMD analysis

**Fig. 5:**
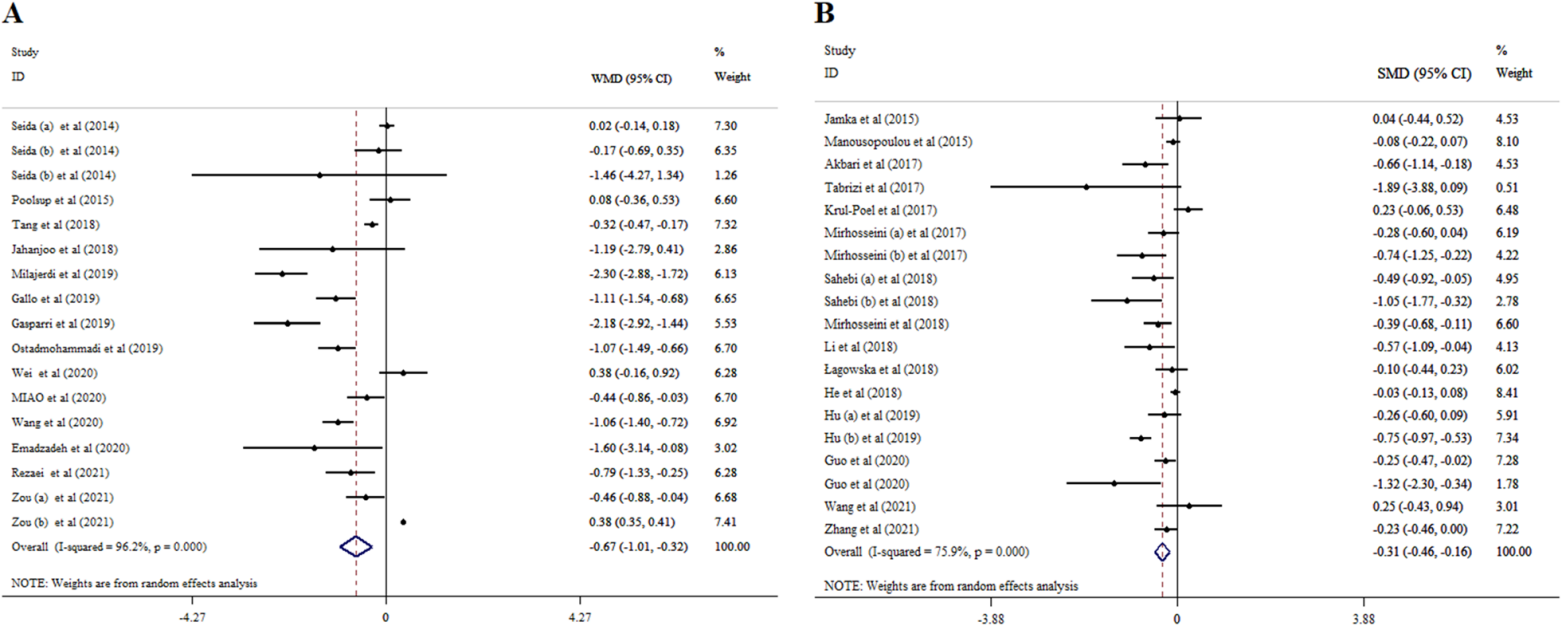
Forest plot with mean difference and 95% confidence intervals (CIs), the effects of vitamin D supplementation on HOMA-IR levels according to WMD (**A**), and SMD (**B**) analysis

The results of 14 meta-analyses with 17 ESs including 47,157 individuals indicated that vitamin D supplementation substantially decreased HOMA-IR (WMD = − 0.67; 95% CI: − 1.01, − 0.32, p < 0.001). The heterogeneity was considerable between studies (*I*^*2*^ = 96.2%, p < 0.001) (Fig. 5A) Vitamin D supplementation resulted in a significant decrease in HOMA-IR at the dosage of ≤ 4000 IU/day, in meta-analyses with intervention duration of ≤ 15 weeks, and those studies that were conducted on women with GDM with sample size ≤ 500 and mean age less than 50 years (Table 3).

#### According to SMD analysis

Vitamin D supplementation decreased HOMA-IR levels (SMD =  − 0.31; 95% CI: − 0.46, − 0.16, p < 0.001, *I*^*2*^ = 75.9%, p < 0.001, 16 meta-analyses with 19 ESs). The *I*^*2*^ index showed considerable heterogeneity among meta-analyses (*I*^*2*^ = 75.9%, p < 0.001) (Fig. 5B). Vitamin D supplementation in a dosage of ≤ 4000 IU/day among > 50 years’ subjects, in studies with intervention duration of ≤ 15 weeks, in patients with GDM, T2DM, and NAFLD, and a sample size of ≤ 500 in women contributed to a more significant reduction in HOMA-IR levels based on the subgroup analyses (Table 4).

### Sensitivity analysis, and publication bias

Stepwise, each study was removed from the analysis to examine the impact of each single meta-analysis on the pooled effect size based on sensitivity analysis. No study significantly changed the total effect size of the study results.

Egger’s and Begg’s tests indicated a small study effect for FBS, HbA1c (only based on WMD analysis), and HOMA-IR (p < 0.05). Moreover, no evidence of a small study effect was detected after conducting Egger’s and Begg’s tests for insulin levels (p˃0.05). Also, visual checking of the funnel plot (Additional file 1: Figs. S1–S4) revealed an asymmetric distribution of included meta-analyses, indicating publication bias. Therefore, trim and fill analysis was carried out, and did not alter the results.

## Discussion

Over the past few decades, a growing body of clinical and epidemiological studies has emerged emphasizing the role of vitamin D on several diseases, such as T2DM, autoimmune disorders, cancer, and cardiovascular disease. In recent years, conflicting findings have been published on the association between circulating serum vitamin D levels with glycemic indices [62, 63]. Therefore, we performed an umbrella review to investigate the available research studies regarding the effect of vitamin D on T2DM biomarkers in adult subjects.

The current umbrella meta-analysis summarized 37 meta-analyses with a total of 36,197 adults. Our analyses shown that vitamin D supplementation significantly decreases FBS, insulin level, HbA1c, and HOMA-IR. Overall, meta-analyses using WMD for reporting the ESs, except HbA1c, revealed a stronger effect than SMD. As WMD depends on the ES of each included meta-analysis, this robust effect was not unexpected. Moreover, in meta-analyses that assessed effect of vitamin D via WMD, we found a greater reduction in FBS in subjects aged > 50 years old, and those with CVD, CKD, and GDM. Also, the vitamin D administrations significantly reduced FBS, insulin, HbA1c, and HOMA-IR at the dosages of ≤ 4000 IU/day compared to > 4000 IU/day, when administered for ≤ 15 weeks. The vitamin D administrations meaningfully reduced insulin, HOMA-IR, and HbA1c at the dosages of ≤ 4000 IU/days, when administered for shorter period of time (≤ 15 weeks). The overall quality of included meta-analyses shown in Table 2 was high to moderate. Publication bias was identified by funnel plot. Nevertheless, this bias did not affect the overall finding identified by trim and fill analysis.

Different parameters such as the latitude, skin pigmentation, duration of sun exposure, and season can affect the production of vitamin D [64, 65]. Several epidemiologic studies propose that low vitamin D levels are related to impaired insulin secretion, insulin resistance, and glucose clearance [66–68]. Also, several previous investigations have shown a relationship between vitamin D deficiency and the progression of T2DM as well as future macrovascular and microvascular complications [69–71]. Our results were consistent with the previous reports, which proposed that vitamin D might help T2DM biomarkers by increasing the absorption of glucose by the improvement of insulin sensitivity [36, 72, 73]. It should be stressed that, however, the results propose that vitamin D supplementation may be efficacious for controlling T2DM biomarkers; the effects of vitamin D on T2DM biomarkers were heterogeneous. Differences between meta-analyses in sample size, population, methodological quality, gender, duration, and dosage may partially explain this heterogeneity. Our subgroup analysis indicated that the effect of vitamin D on T2DM biomarkers was in a time-dependent manner and lower duration of supplementation (≤ 15-weeks) led to a more decrease in T2DM biomarkers in comparison with long term supplementation. There are several reasons which could explain these findings. First of all, it should not be ignored that the 15 weeks period is the time of two seasonal alterations, when the climate conditions and a smaller extent of UV exposure may have an important effect on the production of vitamin D. Besides, daily habits and diet may differ in seasons, which may contribute to the worsening of the metabolic control. Moreover, the participant’s insight of motivation and treatment may have an important effect on the treatment efficacy and mostly long-term intervention decreases the compliance rate. Finally, the fact that the prolonged duration of diseases such as T2DM or gradually worsen with the course of T2DM may help to clarify the result. However, exact interpretation must be with caution since high heterogeneity was observed in both subgroups of sample size and duration. Our study provided evidence proposing that vitamin D supplementation with a dose ≤ 4000 IU/day may be adequate to improve insulin and glucose homeostasis among adults. This is partly because most of the studies used a dose of ≤ 4000 IU/day. Nevertheless, it is possible that vitamin D has favorable effects only in vitamin D deficient participants particularly in those with poor T2DM biomarkers [59, 74].

In our meta-analyses, we observed that vitamin D significantly decreased HbA1c levels, proposing that vitamin D is helpful to delay or decrease the development and occurrence of diabetic problems. In 2007, the UK prospective diabetes study estimated a 1% decrease of HbA1c related to a 14% decrease in risk of cardiovascular events [67]. A review study reported that vitamin D had a helpful effect on glycemic indices in short-term intervention; nevertheless, no significant effect on HbA1c was detected in long term trials with an intervention period > 12 weeks [75]. However, the findings of the current umbrella review indicated that vitamin D was related to a decrease in HbA1c levels in studies with ≤ 15 week's intervention durations. Moreover, there was no significant reduction in FBS, insulin, and HOMA-IR with long-term (> 15 weeks) intervention. Furthermore, the fact of prolonged duration of diseases or gradually worsened condition may help to explain the finding. Moreover, several studies have also revealed that 25(OH) D levels are negatively related to the HOMA-IR and diabetes [76, 77]. The increased HOMA-IR is believed to be caused by the reduced insulin sensitivity. Vitamin D deficiency has been shown to impair insulin secretion in β-cells [78], and Cade et al. [79] propose that improvement of vitamin D status stimulates insulin secretion in rats with vitamin D deficiency. Insulin secretion is a highly dynamic process regulated by several factors such as calcium and hormones [80]. L-type calcium channels on islet β-cells are stimulated by 1, 25(OH) 2D which then controls calcium levels, initiates insulin signaling, and stimulates insulin secretion [80, 81].

The possible mechanisms of action of vitamin D may be through amplification of insulin secretion by the expression of vitamin D (VDR) in the pancreatic β-cells, increasing insulin sensitivity, suppressing the production of pro-inflammatory mediators and cytokines, and regulation of the intracellular and extracellular calcium flux [82–89]. The regulation of insulin secretion is greatly dependent to calcium; therefore, slightly changes in calcium flux can unfavorably affect the secretory role of β-cell [70]. This umbrella of meta-analysis used systematic methods with strong statistical power and robust search strategies, using moderate to high quality researches, which summarized the present literature regarding the effects of vitamin D on T2DM biomarkers. However, our study also has some limitations. Significant between-study heterogeneity detected, which was controlled for, applying subgroup analyses.

## Conclusion

Overall, the present umbrella meta-analysis showed that vitamin D supplementation has lowering effect on FBS, HOMA-IR, HbA1c, and insulin levels. Vitamin D supplementation might be proposed as a beneficial dietary component in managing hyperglycemia and its complications. Moreover, current findings suggest to supplement with a dosage of > 4000 IU and for a treatment period of < 15 weeks. Overall, vitamin D supplementation as a complementary treatment for diabetes management is supported by the findings of this review.

## Supplementary Information

**Additional file 1. **The results of funnel plot for the effect of the vitamin D on glycemic indices.

## Data Availability

Not applicable.
