# Supplementary material for: Effect of vitamin D supplementation on type 2 diabetes biomarkers: an umbrella of interventional meta-analyses
Source: Diabetol Metab Syndr. 2023 Apr 19;15:76. doi: 10.1186/s13098-023-01010-3 (PMC10114333; doi:10.1186/s13098-023-01010-3)
Supplement: Supplementary file 1 — Additional file 1. The results of funnel plot for the effect of the vitamin D on glycemic indices. [file 13098_2023_1010_MOESM1_ESM.docx]

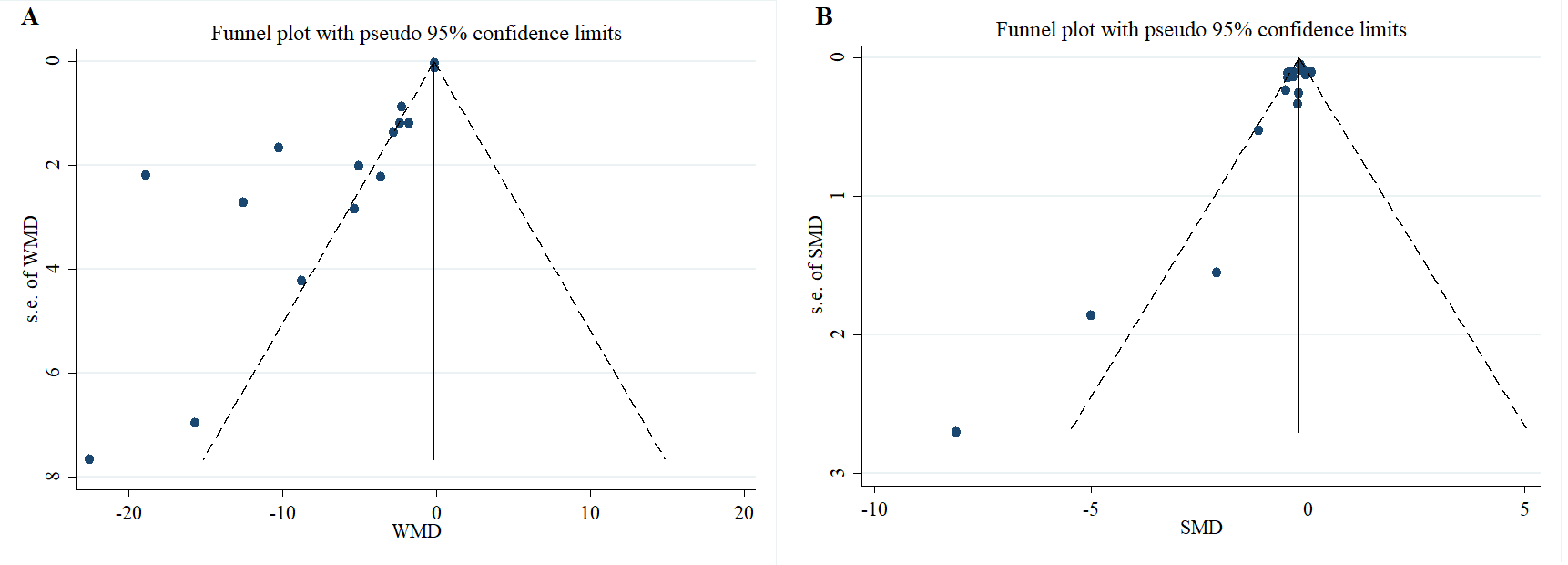

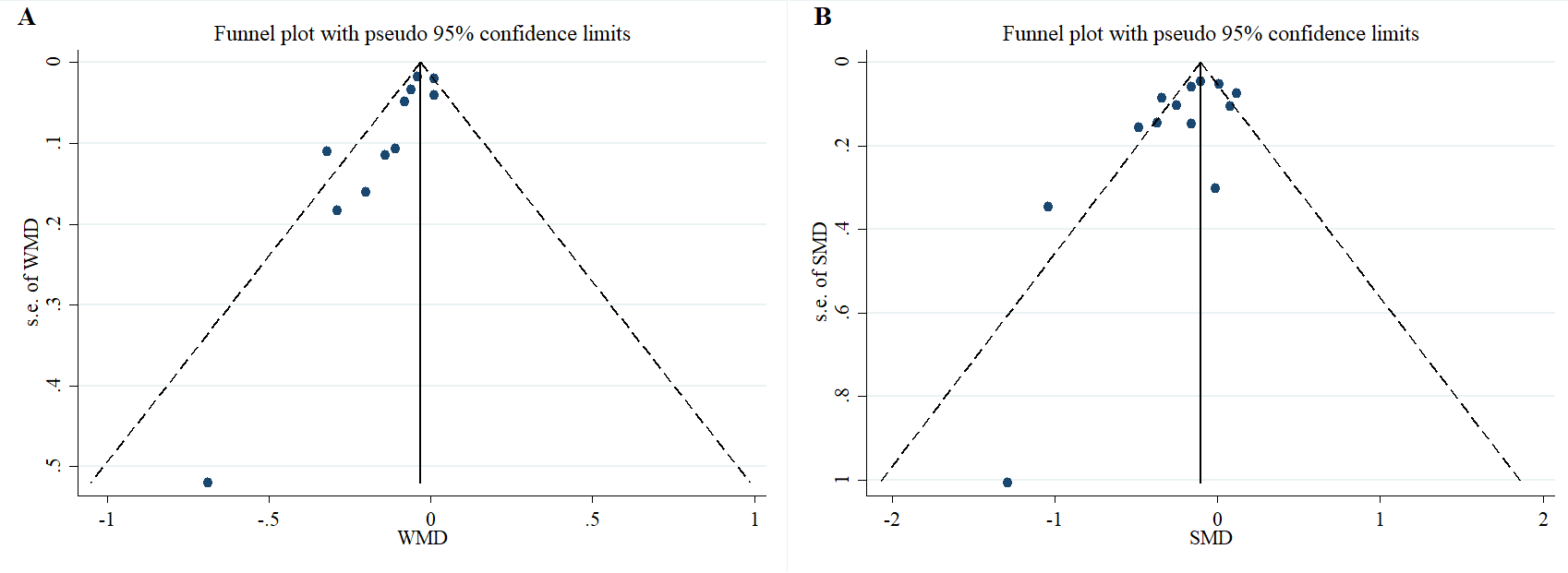

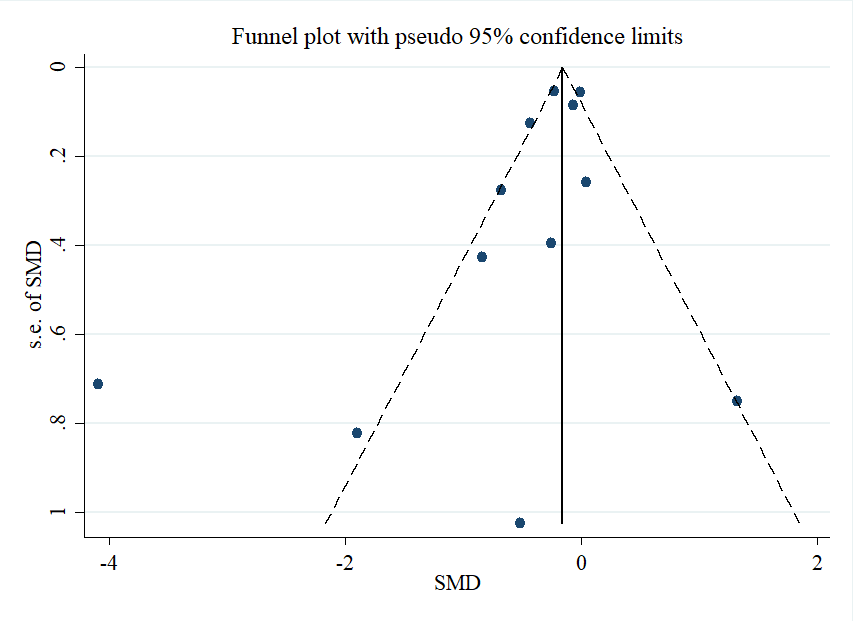

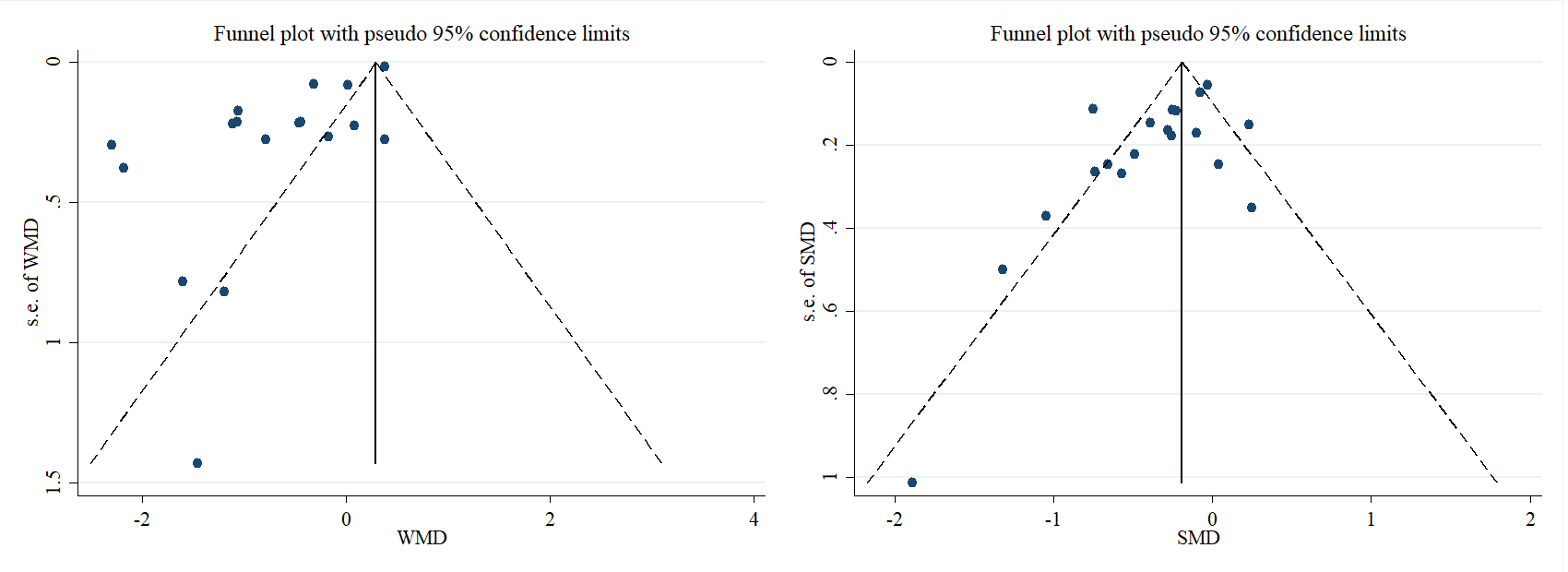


**Figure S1.** Forest plot of the effects of vitamin D supplementation on FBS levels according to WMD (A), and SMD (B) analysis.

**Figure S2.** Forest plot of the effects of vitamin D supplementation on HbA1c levels according to WMD (A), and SMD (B) analysis.

**Figure S4.** Forest plot of the effects of vitamin D supplementation on insulin levels according to SMD analysis.

**Figure S4.** Forest plot of the effects of vitamin D supplementation on HOMA-IR levels according to WMD (A), and SMD (B) analysis.

**Figure S1.** The results of sensitivity analysis for body weight

**Figure S2.** The results of sensitivity analysis for BMI

**Figure S3.** The results of sensitivity analysis for WC

**Figure S4.** Forest plot of the effect of flaxseed on body weight

**Figure S5.** Forest plot of the effect of flaxseed on BMI

**Figure S6.** Forest plot of the effect of flaxseed on WC

**Figure S7.** Random-effects meta-regression plots of the association between sample size, dose, duration of intervention of flaxseed, and weighted mean difference of body weight.

**Figure S9.** Random-effects meta-regression plots of the association between sample size, dose, duration of intervention of flaxseed, and weighted mean difference of WC.

**Figure S10.** Non-linear dose-response relations between duration(week) flaxseed supplementation, and absolute mean differences in body weight.

**Figure S11.** Non-linear dose-response relations between duration(week) flaxseed supplementation, and absolute mean differences in BMI.

**Figure S12.** Non-linear dose-response relations between duration(week) flaxseed supplementation, and absolute mean differences in WC.
